# Reversible hemifacial spasm due to neurocysticercosis

**DOI:** 10.4103/0972-2327.56322

**Published:** 2009

**Authors:** Sushil Razdan, K. K. Pandita, Sarla Pandita

**Affiliations:** Department of Medicine, Acharya Shri Chander College of Medical Sciences, Sidhra, Jammu, J&K, India

**Figure 1:**
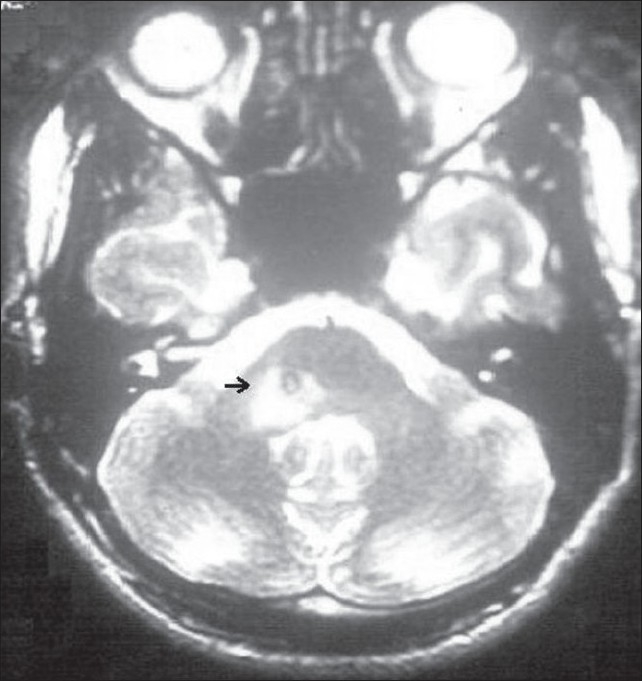
T2 weighted image MRI (axial section) showing an isointense ring lesion with an eccentric hyperintense dot with perilesional edema (arrow)

A 20-year-old man presented with one week history of right facial twitching and involuntary clonic movements. He had acute onset headache, vertigo, imbalance, fever, recurrent vomiting since ten days. These symptoms were very severe and he had received parentral ceftriaxone and dexamethasone. This improved all his symptoms and left him with abnormal right facial movements. There was no history of neck pain or stiffness, altered sensorium, loss of appetite, weight loss, back pain, cough, and night sweating. His past history was unremarkable. Magnetic resonance imaging (MRI) scan of brain with gadolinium enhancement showed, an isointense ring lesion with an eccentric scolex with perilesional edema in right posterior pons at the level of exit of facial and acoustic nerves in the region of seventh right nerve nucleus [Figure 1]. After iv contrast, lesion showed a nodular enhancement with two nodules coalescing. Routine tests were remarkable for peripheral eosinophilia. On reexamination, we noted a subcutaneous nodule in the neck. On excision and subsequent histopathological examination it was reported as cysticercus. We treated the patient with albendazole for eight days and with prednisolone for 14 days. His symptoms resolved completely. He could not afford a follow-up MRI and never reported back after discharge.

Hemifacial spasm (HFS) is one of the motor disorders affecting the muscles of the face supplied by the facial nerve. It consists of a unilateral disturbance affecting, the facial muscles, and producing irregular clonic or twitching movements of the facial muscles, usually of insidious onset. It is thought to arise by synchronous intermittent firing of multiple motor units, with generator being located proximally in the facial nerve.[1] Usually it appears without obvious cause. But sometimes it may be symptomatic of obvious facial nerve compression in brain-stem or at its exit from brain-stem. For example, it can arise as: sequelae in Bell's palsy, multiple sclerosis, guillain-barre syndrome (GBS), or can arise due to cerebello-pontine angle tumor or an aberrant artery that compresses the nerve, or a basilar artery aneurysm, or due to Paget's disease causing compression of facial nerve by bony overgrowth.[2,3] Manifestations of neurocysticercosis are due to mass effect, inflammatory response, or obstruction of brain foramina and ventricular system. Neurological findings are the variables determined by number and location of cysts. Seizures and headaches are the most common presenting features of parenchymal cysts. Others include focal neurological deficits, intracranial hypertension, altered mental status, parkinsonism, cranial nerve palsies, cord compression. Rarely, extensive acute spread of cysticerci to the brain parenchyma can result in cysticercotic encephalitis.[4,5] To our knowledge, neurocysticercosis presenting as seventh nerve motor disturbance (hemifacial spasm or facial myokymia), has not been described in the literature. Neuroimaging findings suggestive of neurocysticercosis constitute major diagnostic criterion. These findings include cystic lesions containing a characteristic scolex with or without enhancement.[6] Neurocysticercosis represents a potentially curable cause of hemifacial spasm or facial myokymia, since neurocysticercosis is endemic in our country. The parenchymal neurocysticercosis is treated with combination of albendazole and prednisolone.[4,5]
